# Prominent response with helical tomotherapy in recurrent ameloblastic carcinoma of maxillary sinus: a case report

**DOI:** 10.1186/1748-717X-9-157

**Published:** 2014-07-15

**Authors:** Timur Koca, Hamit Başaran, Deniz Arslan, Duygu Sezen, Zümrüt Arda Çerkeşli, Özlem Kılınç, Sibel Karaca, Cumhur İbrahim Başsorgun, Hilmi Önder Okay, Münir Demirci

**Affiliations:** 1 Department of Radiation Oncology, Regional Training and Research Hospital, Erzurum, Turkey; 2 Department of Medical Oncology, Regional Training and Research Hospital, Erzurum, Turkey; 3 Department of Pathology, Akdeniz University Medical Faculty Hospital, Antalya, Turkey; 4 Regional Training and Research Hospital, Clinic of Neurosurgery, Erzurum, Turkey; 5 Regional Training and Research Hospital, Clinic of Nuclear Medicine, Erzurum, Turkey

**Keywords:** *Ameloblastic Carcinoma*, *Helical Tomotherapy*

## Abstract

**Introduction:**

Ameloblastoma is a benign but locally aggressive tumor of odontogenic epithelial tissue. Reports of radiotherapy treatment modalities are limited in the literature.

**Case presentation:**

A thirty-five year old male presented with complaints of headache radiating to his face for about six months and impaired vision. The patient’s Positron Emission Tomography (PET) showed a mass in the left maxillary sinus extending to the nasal cavity and invading the adjacent tissues. An R2 (macroscopic residual tumor) surgical resection performed to debulk the tumor. Due to the recurrence and residual mass, the patient was treated with helical tomotherapy. At 2 months post-radiotherapy, patient’s vision returned to normal. PET scan showed a significant reduction in lesion size 12 months post-radiation.

**Conclusion:**

In cases of ameloblastic carcinoma with, post-surgical recurrence or patients not suitable for surgical treatment, helical tomotherapy can be an effective treatment option.

## Introduction

Ameloblastoma is a locally aggressive benign tumor derived from the odontogenic epithelial tissues [1]. The tumor has a slight increased preponderance in females and is mainly diagnosed in the third or fourth decade of life [2,3]. It accounts for about 1% of all jaw tumors [4]. Ameloblastoma prevalently occurs in the ramus and the angulus of the mandible, and rarely in the maxilla [5]. While often clinically asymptomatic, the tumor is usually spotted with bone expansion or detected in routine radiological studies [6]. Numerous histological types have been reported based on the histological findings [7]. The most recent WHO classification has categorized ameloblastoma, to malignant ameloblastoma and ameloblastic carcinoma. Malignant ameloblastoma is different from ameloblastoma since metastases may occur in the former. Both have benign histology. Ameloblastic carcinoma has malignant cytological features regardless of the metastasis occurrence. In ameloblastoma, metastasis rarely occurs [8].

The basic form of treatment for localized ameloblastoma is radical surgery. The only treatment option in metastatic disease appears to be chemotherapy, although the outcome is not favorable. Radiotherapy modalities are limited in the literature [9].

Here we report a case of ameloblastic carcinoma with basal cell histology, where helical tomotherapy achieved a prominent response.

### Case

**Figure 1:**
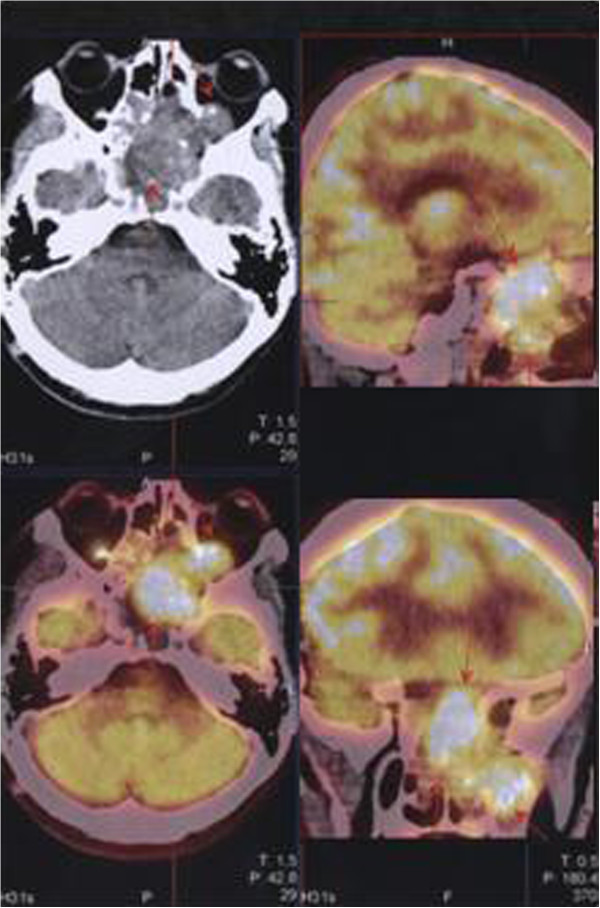
Pre-treatment Positron Emission Tomography (PET) images of the case patient.

**Figure 2:**
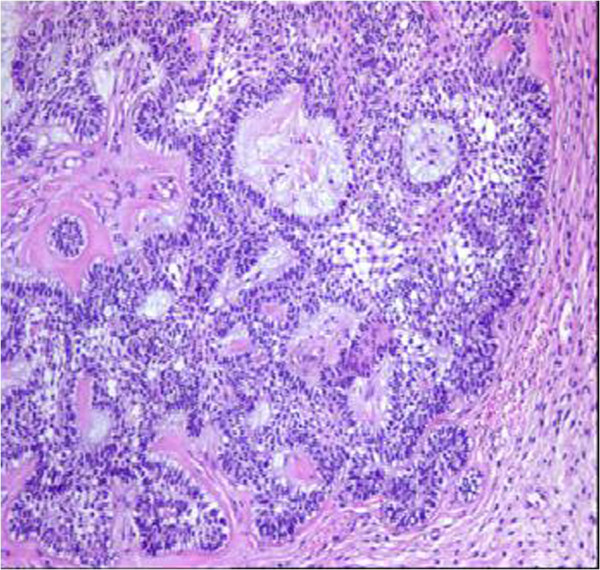
Ameloblastic carcinoma with basaloid appearance.

A thirty-five year old male presented with complaints of headache radiating to his face for about six months and impaired vision. He presented to an outpatient of Ear, Nose and Throat clinic in April 2012. The Magnetic Resonance Imaging (MRI) and Positron Emission Tomography (PET) images (Figure 1) shows a tumor extending to the sphenoidal sinus from the anterior segment of the sphenoid bone. The tumor invades the ethmoid bone, sellar and supra-sellar regions, bilateral retro-orbital areas, almost completely invading the left maxillary sinus and extending to the nasal cavity. The mass was observed to displace optic nerves bilaterally. The tumor size was measured at 57 × 56 × 63 mm. Post-contrast series demonstrated intense and heterogeneous contrast enhancement. Based on the biopsy findings, the patient was diagnosed with ameloblastic carcinoma with basaloid appearance (Figure 2). The patient underwent surgery in the Neurosurgery Clinic in May 2012. Due to the size of the mass and proximity to critical organs, only R2 (macroscopic residual tumor) resection could be performed. The patient developed post-operative infection, and was treated with appropriate antibiotic therapy, which failed to treat the infection. The patient was operated again in June 2012. After the recovery from infection, due to the recurrent and residual mass, the multi-disciplinary oncology council decided to consult with the Radiation Oncology Clinic for post-operative external radiotherapy. The visual field examination The visual assessment conducted by the Eye Diseases Clinic before the initiation of external radiotherapy revealed bilateral visual impairment and diplopia. Serial tomographic sections were taken for the purpose of contouring in radiation therapy plan. The sections were transmitted to the contouring work station through Digital Imaging and Communications in Medicine (DICOM), and Reconstructive Digital Radiography (DRR) was obtained. The DRR contouring of the patient was performed using a Tomocon (Tetramed^TM^, Slovak republic) contouring workstation. The patient’s eyes, optic nerves, lenses, brain stem, optic chiasm, parotid glands, spinal cord, cochleae, vestibules and skin were contoured as critical organs. Prior to the radiotherapy for ameloblastic carcinoma, two separate clinical target volumes (CTV) were defined. CTV1 was defined by adding a 5 mm margin to the gross tumor volume, while planning target volume (PTV) 1 was obtained by adding a 3 mm margin to the CTV1 volume, and then administration of 60 Gy total dose was planned using simultaneous integrated boost (SIB) technique. PTV_2_ was defined by adding a 15 mm margin to CTV_1_ volume, and the prescription dose was 50 Gy to PTV_2_ (Table 1). The patient’s treatment plan was designed with the Tomotherapy planning system (Accuray Inc., Madison, USA). With this planning system, the appropriate prescription doses for organs at risk (OAR) were defined, and routine quality assurance for the prescribed doses was conducted to prepare the patient for the treatment. Related to metastases and close proximity of OAR’s to PTV’s, some of the OAR dose objectives were slightly exceeded Quantec recommendations. The patient was informed about the treatment related adverse effects and informed consent was signed before the start of the treatment session. Prior to each session of treatment, daily Mega Voltage Computerized Tomography (MVCT) scans were performed, and these were compared with the images of treatment planning to achieve set-up accuracy.

**Figure 3:**
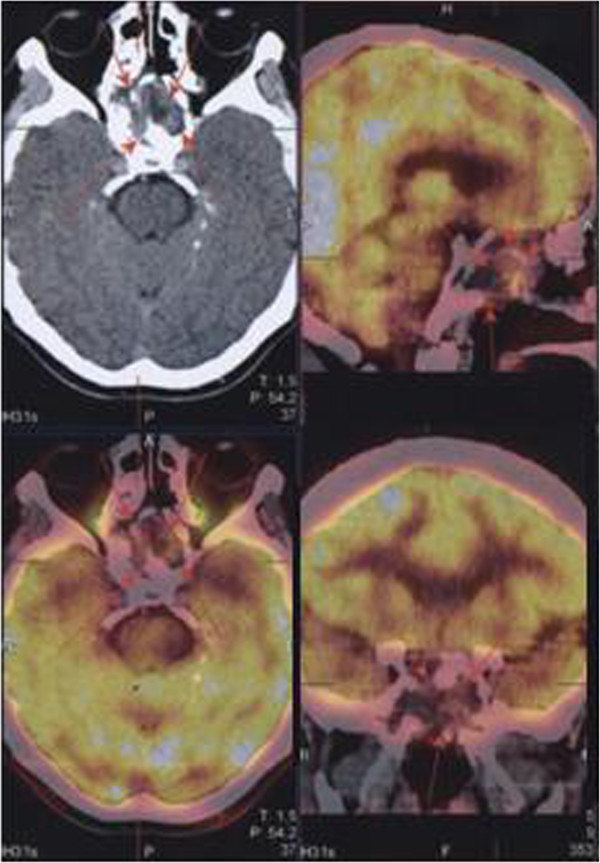
Positron Emission Tomography (PET) images of the patient after external radiotherapy.

The MR images taken at 2 months after radiotherapy showed partial regression in lesion size. Complete clinical remission was achieved in patient’s bilateral visual impairment and diplopia during follow-up. The PET scan taken at 12 months after radiotherapy demonstrated regression of the tumor in the left maxillary sinus, which reduced to 3 × 3.5 × 3 cm, and 18F-fluoro-2-deoxy-D-glucose (FDG) uptake decreased (Previous SUD: 25.36; Current SUD 5.33), while a complete response was achieved in the lesion intracranial extensions (Figure 3). The brain MRI detected no pathological contrast involvement in this area. The patient is under ongoing follow-up. He has not reported any complaints in the check-up conducted in January 2014.

**Table 1:** **Dosimetric Parameters of PTV**_**60**_ (**SIB**) **and PTV**_**54**_

**Variable**	**D**_**max **_**(Gy)**	**D**_**mean **_**(Gy)**	**D**_**min **_**(Gy)**	**HI**	**CI**
***PTV***_***60***_	*64.72*	*61.38*	*47.44*	0.08	0.78
***PTV***_***54***_	64.72	60.45	24.61	0.28	0.82

*Abreviations*: *D*_*max*_ maximum dose, *D*_*mean*_ mean dose, *D*_*min*_ Minimum dose, *PTV* planned target volume, *HI* Homogenity index, *CI* Conformity index.

## Discussion

Although the term ‘ameloblastoma’ was coined by Churchill in 1933, the first detailed description of this lesion was given by Falkson in 1879 [3]. While known as benign odontogenic tumors, ameloblastomas are slow-growing tumors with a high recurrence rate and tendency for local invasion, expansion and destruction in the bone [10-12]. Dental caries, trauma, infection, inflammation, dental disorders, malnutrition and viral pathogens have been suggested to play a role in the etiology [13]. The most common symptom is a slow-growing painless swelling, and less frequently, dental malocclusion, pain, paresthesia or anesthesia might occur. In rare cases, pain may be experienced especially when the tumor is infected, but it causes no symptoms unless there is nerve involvement [6,14].

In 80% of the cases, ameloblastoma originates in the mandible. It mainly involves the angulus and ramus regions of the mandible (70% of cases), whereas 20% of the all cases it involves the premolar region, and 10% the anterior region [2,5,15].

According to the histological findings, the tumors are classified as follicular, plexiform, acanthomatous, granular, basal cell and desmoplastic type [7]. The main clinicoradiographic types of ameloblastoma have been defined as conventional solid or multicystic intraosseous, well-defined unicystic (intraosseous) and peripheral (extraosseous) [10,11,16].

In the diagnosis of ameloblastoma, the radiological tools such as panoramic radiography, CT, MRI and PET-CT can be used. Panoramic radiography is often used in daily practice, while the other methods are better at detecting the presence of metastases, contours, content and soft tissue extension of the lesion [4,5,17,18].

Although considered a benign tumor, ameloblastoma may develop recurrence after resection and become clinically more aggressive, while leading to massive local destruction and metastasis [7]. 15-25% of the cases develop recurrence after radical surgery, while conservative surgery recurrence rate is 75-90% [19]. In patients allowing radical surgery, despite controversies, 1–2 cm margin is sufficient as it significantly decreases the recurrence rate [2]. Since there is always risk of recurrence, even 25–30 years after the primary treatment, patients should be monitored for a long time [3]. Its metastatic spread incidence has been reported as 1 to 4.5% of all cases [2]. Even though rare, cases with metastases to lungs, pleura, spleen, kidney, heart, skull, spine, brain, and lymph nodes have been reported [11]. Surgery may be an option in the presence of metastases. A review of the current literature reveals that various chemotherapeutic agents have been used, including cisplatin, cyclophosphamide, carboplatin, paclitaxel, doxorubicin, methotrexate, prednisone, bleomycin, 5-fluorouracil and dacarbazine, and varying degrees of responses have been reported for each agent [8,20].

As ameloblastoma is a rare and slow-growing tumor, the use of radiotherapy in the treatment should be discussed. Also there are limited data for detailed radiotherapy field design and dose prescriptions. The information regarding radiosensitivity in the current literature is ambiguous [9]. In incomplete resection cases, adjuvant radiotherapy may be considered a treatment option [8,21]. The current studies found in the literature fail to provide sufficient information on the use of radiotherapy/chemoradiotherapy in metastatic disease [8]. Besides, the use of radiotherapy might increase the incidence of conventional bone complications, osteonecrosis and bone carcinoma [9]. With the help of advanced radiotherapy techniques, such complications are tried to be minimized. However, there has been no research examining the use of helical tomotherapy in the treatment of ameloblastoma.

## Conclusion

Ameloblastoma is a slow-growing tumor with no standard chemotherapy treatment options, and primarily treated with curative surgical procedure. In conclusion, we suggest that helical tomotherapy can provide an effective treatment option in ameloblastoma cases where complete resection is not feasible or in patients developing local recurrence.

## Competing interests

The authors declare that they have no competing interests.

## Authors’ contributions

**TK (Corresponding author):** Carried out contouring the patient**,** drafted, generally evaluated and write the manuscript, HB: Made the tables and carried out coordination between clinics, DA: Carried out the patient interrogation and follow-up, write the manuscript, DS: Evaluated the patients radiographs and made calculations, ZAÇ: Carried out the patient set-up preperation, ÖK: Carried out set-up verifications, SK: Prepared the treatment plans of the case, CİB: Carried out histo-pathological examination, HÖO: Carried out surgery, MD: Carried out PET scans. All authors read and approved the final manuscript.
